# Reshaped commensal wound microbiome via topical application of *Calvatia gigantea* extract contributes to faster diabetic wound healing

**DOI:** 10.1093/burnst/tkae037

**Published:** 2024-09-02

**Authors:** Xiaotong Ding, Chenxi Yang, Yue Li, Tangtang He, Yan Xu, Xuxi Cheng, Jinyun Song, Nannan Xue, Wen Min, Weimeng Feng, Hongyu Zhao, Jie Dong, Pei Liu, Yiwei Wang, Jun Chen

**Affiliations:** Jiangsu Provincial Engineering Research Center of TCM External Medication Development and Application, Nanjing University of Chinese Medicine, 138 Xianlin Road, Nanjing 210023, P.R. China; School of Pharmacy, Nanjing University of Chinese Medicine, 138 Xianlin Road, Nanjing 210023, P.R. China; Jiangsu Provincial Engineering Research Center of TCM External Medication Development and Application, Nanjing University of Chinese Medicine, 138 Xianlin Road, Nanjing 210023, P.R. China; Department of Immunology, School of Medicine, Nanjing University of Chinese Medicine, 138 Xianlin Road, Nanjing 210023, P.R. China; Jiangsu Provincial Engineering Research Center of TCM External Medication Development and Application, Nanjing University of Chinese Medicine, 138 Xianlin Road, Nanjing 210023, P.R. China; School of Pharmacy, Nanjing University of Chinese Medicine, 138 Xianlin Road, Nanjing 210023, P.R. China; Jiangsu Provincial Engineering Research Center of TCM External Medication Development and Application, Nanjing University of Chinese Medicine, 138 Xianlin Road, Nanjing 210023, P.R. China; School of Pharmacy, Nanjing University of Chinese Medicine, 138 Xianlin Road, Nanjing 210023, P.R. China; Department of Bone injury of Traditional Chinese Medicine, Affiliated Hospital of Nanjing University of Chinese Medicine, 155 Hanzhong Road, Nanjing 210029, P.R. China; Jiangsu Provincial Engineering Research Center of TCM External Medication Development and Application, Nanjing University of Chinese Medicine, 138 Xianlin Road, Nanjing 210023, P.R. China; School of Pharmacy, Nanjing University of Chinese Medicine, 138 Xianlin Road, Nanjing 210023, P.R. China; Jiangsu Provincial Engineering Research Center of TCM External Medication Development and Application, Nanjing University of Chinese Medicine, 138 Xianlin Road, Nanjing 210023, P.R. China; School of Pharmacy, Nanjing University of Chinese Medicine, 138 Xianlin Road, Nanjing 210023, P.R. China; Clinical Research Center, The Second Hospital of Nanjing, Nanjing University of Chinese Medicine, 1 Kangfu Street, Nanjing 210003, P.R. China; Jiangsu Provincial Engineering Research Center of TCM External Medication Development and Application, Nanjing University of Chinese Medicine, 138 Xianlin Road, Nanjing 210023, P.R. China; School of Pharmacy, Nanjing University of Chinese Medicine, 138 Xianlin Road, Nanjing 210023, P.R. China; Department of Bone injury of Traditional Chinese Medicine, Affiliated Hospital of Nanjing University of Chinese Medicine, 155 Hanzhong Road, Nanjing 210029, P.R. China; School of Pharmacy, Nanjing University of Chinese Medicine, 138 Xianlin Road, Nanjing 210023, P.R. China; Clinical Research Center, The Second Hospital of Nanjing, Nanjing University of Chinese Medicine, 1 Kangfu Street, Nanjing 210003, P.R. China; Clinical Research Center, The Second Hospital of Nanjing, Nanjing University of Chinese Medicine, 1 Kangfu Street, Nanjing 210003, P.R. China; Jiangsu Provincial Engineering Research Center of TCM External Medication Development and Application, Nanjing University of Chinese Medicine, 138 Xianlin Road, Nanjing 210023, P.R. China; School of Pharmacy, Nanjing University of Chinese Medicine, 138 Xianlin Road, Nanjing 210023, P.R. China; School of Pharmacy, Nanjing University of Chinese Medicine, 138 Xianlin Road, Nanjing 210023, P.R. China; Jiangsu Collaborative Innovation Center of Chinese Medicinal Resources Industrialization, Nanjing University of Chinese Medicine, 138 Xianlin Road, Nanjing 210023, P.R. China; Jiangsu Provincial Engineering Research Center of TCM External Medication Development and Application, Nanjing University of Chinese Medicine, 138 Xianlin Road, Nanjing 210023, P.R. China; School of Pharmacy, Nanjing University of Chinese Medicine, 138 Xianlin Road, Nanjing 210023, P.R. China; Jiangsu Collaborative Innovation Center of Chinese Medicinal Resources Industrialization, Nanjing University of Chinese Medicine, 138 Xianlin Road, Nanjing 210023, P.R. China; Jiangsu Provincial Engineering Research Center of TCM External Medication Development and Application, Nanjing University of Chinese Medicine, 138 Xianlin Road, Nanjing 210023, P.R. China; School of Pharmacy, Nanjing University of Chinese Medicine, 138 Xianlin Road, Nanjing 210023, P.R. China; Jiangsu Collaborative Innovation Center of Chinese Medicinal Resources Industrialization, Nanjing University of Chinese Medicine, 138 Xianlin Road, Nanjing 210023, P.R. China

**Keywords:** Calvatia gigantea extract, Diabetic wound healing, Commensal wound microbiome, Macrophage polarization, Inflammation

## Abstract

**Background:**

*Calvatia gigantea* (CG) is widely used as a traditional Chinese medicine for wound treatment. In this study, we aimed to determine the effects of CG extract (CGE) on diabetic wound healing and the commensal wound microbiome.

**Method:**

A wound model was established using leptin receptor-deficient db/db mice, with untreated mice as the control group and CGE-treated mice as the treatment group. The wound healing rate, inflammation and histology were analyzed. Additionally, wound microbiome was evaluated via 16S ribosomal RNA (rRNA) gene sequencing.

**Results:**

CGE significantly accelerated the healing of diabetic ulcer wounds, facilitated re-epithelialization, and downregulated the transcription levels of the inflammatory cytokines, interleukin-1β and tumor necrosis factor-α. Furthermore, CGE treatment positively affected the wound microbiome, promoting diversity of the microbial community and enrichment of *Escherichia–Shigella* bacteria in the CGE-treated group.

**Conclusions:**

Overall, CGE enhanced diabetic wound healing by modulating the wound microbiome and facilitating macrophage polarization during inflammation. These findings suggest modulation of the commensal wound microbiome using medicinal plants as a potential therapeutic strategy for diabetic wounds.

## Graphical Abstract

**Graphical Abstract:**
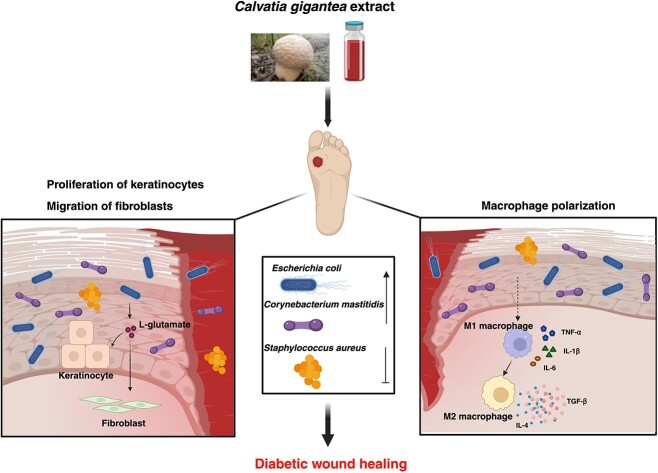

Highlights

- CGE significantly accelerated the healing rate of diabetic ulcer wounds, facilitated re-epithelialization, and downregulated the transcription levels of inflammatory cytokines interleukin (IL)-1β and tumor necrosis factor-α (TNF-α)
- CGE could reduce the abundance of pathogenic bacteria such as *Staphylococcus aureus* and significantly enrich *Escherichia coli (E. coli)* by regulating the microecology of diabetic ulcer bacteria
- *E. coli* can increase the secretion level of L-glutamate under CGE treatment, which promotes the cell proliferation and migration level of keratinocytes and fibroblasts as well as epithelialization and tissue barrier regeneration
- Modulation of commensal wound microbiome may be a new therapeutic strategy for the treatment of diabetic wound repair

## Background

More than 550 million people worldwide were estimated to have diabetes in 2023 [1]. Diabetic ulcers (DUs) are common high-risk complications in patients with diabetes. Approximately 34% of patients with type 1 or type 2 diabetes develop DUs during their lifetime [2]. Approximately 20% of patients with DUs undergo lower limb amputation of a part of the foot (minor) or the entire foot (major) [3]. High incidence, amputation, recurrence and mortality rates seriously affect the quality of life of patients with DUs [2]. Due to the complex pathogenesis of DUs, their effective treatment is challenging in clinical practice. Primary clinical treatments for DUs include surgical skin grafting, skin flap transplantation, cell therapy, growth factor treatment and bioengineering [1,4]. However, these treatments have many limitations, including long hospital stay, high cost and many confounding factors. The increasing aging population and diabetes prevalence worldwide along with the absence of effective clinical approaches necessitate the development of new drugs and treatment strategies for DUs [5].

Skin is the external interface between the human body and the environment; it is a physical barrier that prevents foreign pathogens from invading the body and consists of millions of bacteria, fungi and viruses [6]. Surface flora play an important role in human health [7] and are known as the ‘guardians of skin health’ [8]. Wound microorganisms regulate host inflammatory responses [9]. Skin symbiosis accelerates wound healing [10] and induces skin regeneration [11]. However, decreased angiogenesis caused by hyperglycemia and dysregulation of the immune response disturb the bacterial flora in DUs, which can be improved by regulating the microflora in diabetic wounds [12]. Moreover, because of hyperglycemia, poor angiogenesis, reduced reactive oxygen species levels and excessive free radicals, pro-inflammatory cytokines are overexpressed in diabetic wounds, resulting in inadequate formation of the extracellular matrix [13], decreased cell proliferation [14], and low migration of keratinocytes, fibroblasts and vascular endothelial cells [15]. A key step in diabetic wound healing is the transition from the inflammatory to the proliferative stage [16]. Macrophages play a key role in the inflammatory stage of healing, in which they are polarized into pro-inflammatory M1 or anti-inflammatory M2 macrophages [17]. Impaired M1-to-M2 macrophage conversion is closely associated with delayed wound healing, poor angiogenesis and reduced collagen deposition in diabetic wounds [18].

*Calvatia gigantea* (Batsch ex Pers.) Lloyd (CG) is a fungus used in medicine and food. It contains phenolic compounds [19] and sugars [20] and exerts sanitary [21] and antioxidant effects [22]. CG is widely used for wound healing and prevention of bleeding via topical administration in traditional Chinese medicine [23,24]. We previously reported that CG spore powder accelerates diabetic wound healing by promoting the Akt/nuclear factor E2-related factor 2 pathway and antioxidant gene expression [25]. CG extract (CGE) exerts antioxidant, antidiabetic [26] and antitumor effects [27]. In this study, we investigated the roles of CGE in diabetic wound healing using a db/db mouse injury model. Furthermore, we conducted 16S ribosomal RNA (rRNA) gene sequencing, metagenomic and metabolomic analyses to explore the effects of CGE on host microbial species and key metabolites to promote wound healing.

## Methods

### Preparation of CGE

CG was purchased from (blind for peer review) and authenticated by (blind for peer review) based on the Pharmacopoeia of the People’s Republic of China (Part I, 2020 version). Its outer shell was crushed and the impurities were removed. CG spore powder was obtained after passing it thrice through a filter screen. Subsequently, the powder was immersed in 95% ethanol for 24 h at a solid–liquid ratio of 1 : 15 and ultrasonic extraction was performed for 2 h (45 kHz, 30°C) to collect the supernatant. The supernatant was evaporated and lyophilized to obtain the final CGE product. The CGE powder was stored at −20°C. Voucher specimens of CGE (S20210003, 20 210 011 and 20 210 021) have been deposited in our laboratory.

### Ultra-high performance liquid chromatography-quadrupole time-of-flight tandem mass spectrometry analysis of CGE

CGE was dissolved in ethanol to obtain a solution. Then, CGE solution (10 g/ml) was diluted thrice with ethanol and 1 ml of the diluent was added to the pre-activated solid-phase extraction column. Next, 2 ml each of 5, 25, 50, 75 and 100% methanol solutions were used for gradient elution. The solution was centrifuged at 13,000 rpm for 10 min at 4°C and the supernatant was collected as the CGE test product solution.

**Chromatographic conditions.** An Agilent EXTEND-C18 column (100 × 2.1 mm, 1.8 μm) was used. Column temperature: 40°C; mobile phase: 0.1% formic acid water (A)–acetonitrile (B); flow rate: 0.3 ml/min; sample volume: 1 μl; duration: 32 min. Gradient elution was performed as presented in Table 1.

**Table 1:** Mobile-phase gradient elution procedure

**Time/min**	**A/%**	**B/%**
0.01	95	5
26.00	5	95
28.00	5	95
30.00	95	5
32.00	95	5

**Mass spectrometry conditions.** For quadrupole time-of-flight tandem mass spectrometry a Q-TOF-MS/MS 5600^+^ mass spectrometer and electrospray ionization source were used for high-sensitivity scanning in positive- and negative-ion modes. Primary mass spectrum data acquisition parameters are listed below. Mass scanning range (*m*/*z*): 100–2000; cumulative time: 0.15 s; auxiliary gas: nitrogen; atomizing gas (GS1): 55 psi (1 psi ≈ 6.895 kPa); auxiliary gas (GS2): 55 psi; air curtain gas (CUR): 35 psi; ionization temperature (TEM): 550°C; ion spray voltage (IS): +5500/−4500 V; decluster voltage: +/−60 V; collision voltage: +/−10 eV. Secondary mass spectrum data acquisition parameters are listed below. Mass scanning range (*m*/*z*): 50–1500; cumulative time: 0.05 s; GS1: 55 psi; GS2: 55 psi; CUR: 35 psi; TEM: 550°C; IS: +5500/−4500 V; decluster voltage: +/−60 V; collision voltage: +/−40 eV; collision energy expansion: 20 eV; mass scanning range (*m*/*z*): 100–2000. Raw data was acquired using the Analyst TF 1.6 workstation with the Q-TOF-MS/MS 5600^+^ mass spectrometer.

Original data was imported into the PeakView software (version 1.2; AB Sciex companies, USA) using the open source ChemSpider (http://chemspider.com), PubChem (https://pubchem.ncbi.nlm.nih.gov) and MassBank of North America (https://mona.fiehnlab.ucdavis.edu) databases, and the secondary spectra of fragments were identified. [M + H]^+^, [M + Na]^+^, [M + K]^+^, [M + NH_4_]^+^, [2 M + H]^+^ and other adduct modes were considered as the positive-ion modes, whereas [M-H]^−^, [M]-H_2_O-H]^−^, [M + FA-H]^−^, [2 M-H]^−^, [M-2H]^2−^, [M + Na-H]^−^ and other adduct modes were considered as the negative-ion modes [error (ppm): ≤ | ± 25|]. According to the score of the compounds preliminarily identified using the software, compounds with scores >80 were further manually screened to detect false positives. Original data were imported into the Markerview software for retention time correction, peak matching and peak alignment. MS1 error was set to <0.015 Da, and the corresponding identified peak area and other data were saved for subsequent analysis. The total ion chromatogram of the CGE components is shown in Figure S1 (see online supplementary material), and identification information for the CGE compounds is presented in Table S1 (see online supplementary material).

### CGE concentration screening

Mouse skin fibroblasts (MSFs) were purchased from Shanghai Fuheng Biotechnology Co., Ltd. MSFs were cultured in high-glucose Dulbecco’s modified Eagle’s medium, containing 100 U/ml penicillin, 100 mg/l streptomycin and 15% v/v fetal bovine serum, in a constant temperature incubator at 37°C and 5% CO_2_. Cells were passaged every 2–3 days and those in the logarithmic stage were used for subsequent experiments. MSFs at passages 2–4 were seeded at a density of 5000–8000 cells/well in 96-well plates for 24 h and cultured with various concentrations of CGE (0, 1, 5, 10, 20, 50, 100 and 200 μg/ml). After 24 h, cell viability was assessed using the cell counting kit (CCK)-8 assay and the optimal concentration of CGE to promote MSF proliferation was determined.

### Animal experiment

Male db/db mice (age: 11–12 weeks old; weight: 40–50 g; *n* = 6/group/time point) were purchased from Gempharmatech Co., Ltd (Jiangsu, China) and housed in cages (five per cage) with free access to water in a specific-pathogen-free facility at (blind for peer review). The mice were kept in a controlled environment at 24–26°C and 44–46% humidity under a 12:12 h light/dark cycle.

Before inducing an injury, db/db mice were anesthetized using 3% isoflurane. Hair on the dorsal area of each animal was removed using a mouse clipper and cleaned with betadine solution. A wound measuring 1 cm^2^ (~5% of the total body surface area) was surgically created. All animals were randomly assigned to either the control group (open wounds, wounds coated with saline, 100 μl/wound) or CGE treatment group (wounds coated with CGE, 100 μg/ml, 100 μl/wound), and all mice were treated on days 0, 3, 7 and 14. After wounding, the animals were administered 1 ml of warm resuscitative intraperitoneal saline for 3 days. All db/db mice were housed individually and monitored daily for signs of distress or changes in physical appearance. The wound sizes on days 0, 3, 7, 14 and 21 were imaged and recorded until complete wound closure. Mice were euthanized via cervical dislocation at different time points, and the skin and wound tissues were harvested for histological and mRNA expression analysis. Blood was collected via cardiac puncture before euthanasia. Blood samples were centrifuged at 10,000 rpm, and the serum was isolated and stored at −80°C until further analysis.

### Histological analysis

Wound tissues were fixed with 10% formalin for 24 h and dehydrated using graded concentrations of ethanol and xylene. Subsequently, the wound tissues were embedded in paraffin and sectioned at a thickness of 5 μm. Each tissue section was stained with hematoxylin and eosin (H&E) for general histological analysis and collagen deposition was assessed via Masson’s trichrome staining. Anti-cluster of differentiation (CD) 206 XP rabbit mAbs (1400, CST) were used for immunofluorescence analysis. Histological observations were performed via morphological analysis using an optical microscope (Axio Vert A1; ZEISS). Wound collagen fiber density was measured using ImageJ 2.1.0/1.53c software.

### Measurement of serum and tissue inflammatory cytokine levels

Bio-plex Pro cytokine 23-plex assay (Univ-Bio, China) was used to determine the expression levels of inflammatory mediators, including interleukin (IL)-2, IL-4, IL-5, IL-6, IL-10, interferon-γ (IFN-γ), IL-1β, tumor necrosis factor-α (TNF-α) and keratinocyte-derived cytokine, in terminal blood samples collected at day 7 post-injury. The assay was performed according to the manufacturer’s instructions.

Total RNA was isolated from the wound tissue using the TRIzol reagent (Invitrogen), and cDNA was synthesized using the Prime Script RT system (Takara). After cDNA synthesis, reverse transcription-polymerase chain reaction (RT-PCR) was performed using the Roche LightCycler 96 system to determine the levels of **Il-1β*, *Il-18**, tumor necrosis factor α (*Tnf-α*), *Il-6*, *Cd80*, *Cd86*, nitric oxide synthase 2 (*Nos2*), *Il-10,* arginase 1 (*Arg1*), *Il-1a*, peroxisome proliferator-activated receptor γ (*Pparγ*), transforming growth factor β *(Tgf-β*), *Cd163*, *Il-4*, Toll-like receptor 2 (*Tlr2*), matrix metalloproteinase 9 (*Mmp9*), tissue inhibitor of metalloproteinase 1 (*Timp1*), collagen type I α1 (*Col1 α1*), *Col3 α1*, Fibronectin *(Fn1*), tenascin-c (*Tnc)*, proliferating cell nuclear antigen (*Pcna*), melanoma cell adhesion molecule (*Mcam*) and vascular endothelial growth factor a (*Vegfa*). Actin β (*Actβ*) mRNA was used as an internal control. All primer sequences used in this study are shown in Table 2.

**Table 2:** Sequences of the primers designed for RT-PCR

**Gene**	**Forward primer**	**Reverse primer**
*Il-1β*	GAAATGCCACCTTTTGACAGTG	TGGATGCTCTCATCAGGACAG
*Il-18*	GTGAACCCCAGACCAGACTG	CCTGGAACACGTTTCTGAAAGA
*Tnf-α*	GAGGCCAAGCCCTGGTATG	CGGGCCGATTGATCTCAGC
*Il-6*	GTCCTTCCTACCCCAATTTCCA	TAACGCACTAGGTTTGCCGA
*Cd80*	AAAGCCTCGCTTCTCTTGGT	TCCTCTGACACGTGAGCATC
*Cd86*	CTGGACTCTACGACTTCACAATG	AGTTGGCGATCACTGACAGTT
*Nos2*	GTTCTCAGCCCAACAATACAAGA	GTGGACGGGTCGATGTCAC
*Il-1α*	TCGCAGCAGGGTTTTCTAGG	CAGCTTTAAGGACGGGAGGG
*Pparγ*	TGAAGGCTGCAGCGCTAAAT	CGAGTGGTCTTCCATCACGG
*Tgf-β*	CGTCAGACATTCGGGAAGCA	TGCCGTACAACTCCAGTGAC
*Cd163*	ACCAACGAAGCCCACAAAGA	CCTTTTGGACACTGCACACG
*Arg1*	CTCCAAGCCAAAGTCCTTAGAG	GGAGCTGTCATTAGGGACATCA
*Mrc1*	GTGGGGACCTGGCAAGTATC	CACTGGGGTTCCATCACTCC
*Il-4*	GTACTCGTCCCTCACTTGCC	CTTGGGTGCTGACATGAGGT
*Il-10*	CAAACAGTACGGAAACTCAACCT	GGTGATACAGATCCAGGGTGAAC
*Tlr2*	CACTGGGGGTAACATCGCTT	AGTCAGGTGATGGATGTCGC
*Mmp9*	GGACCCGAAGCGGACATTG	CGTCGTCGAAATGGGCATCT
*Timp1*	AGAGTGTCTGCGGATACTTCC	CCAACAGTGTAGGTCTTGGTG
*Col1α1*	CTGGCGGTTCAGGTCCAAT	TTCCAGGCAATCCACGAGC
*Col3α1*	CTGTAACATGGAAACTGGGGAAA	CCATAGCTGAACTGAAAACCACC
*Fn1*	ATTGCCCCATTGAGTGCTTC	ATTGTGCTGAAGCTGAGAACTAGG
*Tnc*	CTTGGCTTGTCAGGGCTTGTCC	AGACCACTGCGCTCCACGTTGA
*Pcna*	TGTGCCCCTTGTTGTAGAGT	AAAGACCTCAGGACACGCTG
*Mcam*	CTTCAGCCAAGTGGACTGGT	GTAGTGATCCTGGAGCCGTG
*Vegfa*	GCAGATGTGAATGCAGACCAA	TCTCCGCTCTGAACAAGGCT
*Actβ*	GGCTGTATTCCCCTCCATCG	CCAGTTGGTAACAATGCCATGT

*RT-PCR* reverse transcription-polymerase chain reaction*, Il-1β* interleukin 1β, *Tnf-α* tumor necrosis factor α, *Cd80* cluster of differentiation 80, *Nos2* nitric oxide synthase 2, *Pparγ* peroxisome proliferator-activated receptor γ, *Tgf-β* transforming growth factor β, *Arg1* arginase 1, *Mrc1* mannose receptor C-type 1, *Tlr2* toll-like receptor 2, *Mmp9* matrix metalloproteinase 9, *Timp1* tissue inhibitor of metalloproteinase 1, *Col1α1* collagen type I α1, *Col3α1* collagen type III α1, *Fn1* fibronectin, *Tnc* tenascin-c, *Pcna* proliferating cell nuclear antigen, *Mcam* melanoma cell adhesion molecule, *Vegfa* vascular endothelial growth factor a, *Actβ* actin β

### *16S rRNA* gene sequencing analysis of the wound microbiome

Wound microbiome samples were collected from db/db mice on days 3 and 14 post-injury via swabbing and sent to Majorbio Bio-Pharm Technology (Shanghai, China) for Illumina *16S rRNA* gene sequencing. Briefly, microbial community genomic DNA was extracted from the wound samples and used to generate *16S rRNA* libraries, as previously described [10]. After PCR, the purified amplicons were pooled in equimolar amounts and paired-end sequenced on an Illumina MiSeq PE300 platform/NovaSeq PE250 platform (Illumina, San Diego, CA, USA) according to the manufacturer’s protocols.

Raw data of *16S rRNA* gene sequencing reads were demultiplexed, quality-filtered using fastp version 0.19.6 and merged using FLASH version 1.2.11 according to the criteria listed below. (1) 300 bp reads were truncated at any site receiving an average quality score < 20 over a 50 bp sliding window, and truncated reads < 50 bp and reads containing ambiguous characters were discarded. (2) Only overlapping sequences >10 bp were assembled according to their overlapping sequence. The maximum mismatch ratio in the overlapping region was 0.2. Reads that could not be assembled were discarded. (3) Samples were distinguished according to the barcode and primers, and sequence direction was adjusted for exact barcode matching and two nucleotide mismatches in primer matching. Operational taxonomic units with a 97% similarity cutoff were clustered using UPARSE version 7.0.1090, and chimeric sequences were identified and removed. The taxonomy of each operational taxonomic unit representative sequence was analyzed using ribosomal database project classifier version 2.11 against the *16S rRNA* database (eg. Silva v138) using a confidence threshold of 0.7. Alpha diversity analysis, principal coordinate analysis (PCoA), species Venn diagram analysis, community bar diagram analysis, intergroup significance difference test and linear discriminant analysis effect size (LEfSe) multistage species difference discriminant analysis were used to analyze the diverse wound microbiome.

### Metagenomic analysis of the wound microbiome

Wound microbiome samples were collected from db/db mice on day 14 post-injury via swabbing and sent to Majorbio Bio-Pharm Technology for metagenomic analysis. Total genomic DNA was extracted from the wound microbiome samples using the E.Z.N.A Soil DNA Kit (Omega Bio-tek, Norcross, GA, USA), according to manufacturer’s instructions. Concentration and purity of the extracted DNA were determined using TBS-380 and NanoDrop2000, respectively. Then, the quality of the DNA extract was checked using 1% agarose gel. The DNA extract was fragmented to an average size of ~400 bp using Covaris M220 (Gene Company Limited, China) for paired-end library construction. A paired-end library was constructed using NEXTflex Rapid DNA-Seq (Bioo Scientific, Austin, TX, USA). Adapters containing the full complement of the sequencing primer hybridization sites were ligated to the blunt ends of the fragments. Paired-end sequencing was performed on the Illumina NovaSeq platform at Majorbio Bio-Pharm Technology Co., Ltd using NovaSeq Reagent Kits (www.Illumina.com).

Raw reads from metagenome sequencing were used to generate clean reads by removing the adaptor sequences and trimming and removing the low-quality reads (reads with N bases, minimum length threshold of 50 bp and minimum quality threshold of 20) using fastp [28] (https://github.com/OpenGene/fastp, version 0.20.0) on the free Majorbio Cloud Platform (cloud.majorbio.com). These high-quality reads were then assembled into contigs using MEGAHIT [29] (parameters: kmer_min = 47, kmer_max = 97, and step = 10; https://github.com/voutcn/megahit; version 1.1.2), which uses succinct de Bruijn graphs. Contigs >300 bp in length were selected from the final assembly results.

Next, open reading frames of the contigs were identified using MetaGene [30] (http://metagene.cb.k.u-tokyo.ac.jp/). Predicted open reading frames with a length of 100 bp were retrieved and translated into amino acid sequences using the National Center of Biotechnology Information translation table (http://www.ncbi.nlm.nih.gov/Taxonomy/taxonomyhome.html/index.cgi?chapter = tgencodes#SG1). A non-redundant gene catalog was constructed using Cluster Database at High Identity with Tolerance [31] (http://www.bioinformatics.org/cd-hit/; version 4.6.1) with 90% sequence identity and 90% coverage. Reads after quality control were mapped to the non-redundant gene catalog with 95% identity using SOAPaligner [32] (http://soap.genomics.org.cn/; version 2.21), and gene abundance in each sample was evaluated. A community heatmap was used to analyze the similarities and differences between different samples in terms of species composition. The Circos sample–species relationship map was used to identify the dominant species in each sample. Species and functional contribution analyses were performed to determine the degree of functional contribution of specific species and functions.

### *In vitro* effects of CGE on the skin-colonizing microbe *Escherichia coli (E. coli)*

*E. coli* (LWCC1033) from Shanghai Weilu Technology Co., LTD. (Shanghai, China) was cultured upside-down on the Luria–Bertani (LB) solid medium at 37°C. In aseptic conditions, the strains were selected with an aseptic inoculation ring, transferred to 50 ml of fresh LB liquid medium and shaken at 37°C for 12–18 h until the optical density (OD; λ = 630 nm) was ~0.8. The activated *E. coli* liquid was transferred to sterile water to dilute the *E. coli* solution to multiples of 10^−4^ and 10^−8^.

**Dilution-plate method** LB solid medium was prepared and autoclaved at 121°C for 15 min and cooled to 50°C. Sterile water or CGE was added to divide the medium into control and CGE group parts (100 μg/ml). After the medium was solidified, 100 μl of *E. coli* diluent at the ratio of 10^−8^ was absorbed, and the dilute solution was spread clockwise with a sterile coating rod until the bacterial solution was evenly distributed; the growth of *E. coli* was observed after 24 h of inverted culture at 37°C.

**Growth curve method** LB liquid medium was prepared and sterile water or CGE was added to divide the medium into control and CGE group parts (100 μg/ml). Then, 1 ml of *E. coli* diluted at the ratio of 10^−4^ was absorbed and shaken continuously at 37°C. Solutions at 0, 6, 12, 24, 36, 48 and 60 h were sampled to determine the OD and plot the growth curve of *E. coli* (λ =630 nm).

### Metabolomic analysis of CGE-treated *E. coli*

*E. coli* from the control and CGE groups (100 μg/ml) were incubated in the liquid LB medium for 36 h. *E. coli* was sharply frozen prior to metabolomic analysis by Majorbio Bio-Pharm Technology. Briefly, metabolomic analysis was conducted using an ultra-high-performance liquid chromatography tandem Fourier transform mass spectrometry UHPLC-Q Exactive HF-X system; Thermo Field). Chromatographic conditions were set as: chromatography on ACQUITY UPLC HSS T3 (100 × 2.1 mm i.d., 1.8 μm, Waters, Milford, USA), mobile phase A was water/acetonitrile (95/5%), mobile phase B was acetonitrile/isopropanol/water (47.5/47.5/5%) and sample size was 2 μl. Positive- and negative-ion scanning modes were used for mass spectrum analysis and the quality scanning range was set at *m*/*z* 70–1050. Ion-spray voltage: +3500/−3500 V; S-lens voltage: 50 V; heating temperature: 425°C; and capillary temperature: 325°C. Raw data processing software, Progenesis QI import metabolomics (Waters), was used to identify the baseline filter, peak, integral, peak retention time correction, alignment, retention time, mass-to-charge ratio, and peak intensity information, such as the data matrix. Then, the software was used to identify the characteristic peak database, mass spectrometry (MS) and tandem MS (MS//MS) mass spectrum information was matched with the metabolic database, MS mass error was set to <10 ppm and metabolites were identified according to the secondary mass spectrum matching score.

Differential volcano maps and an orthogonal partial least squares discriminant analysis (OPLS-DA) model were used to determine the overall differences between the groups of samples. Kyoto Encyclopedia of Genes and Genomes (KEGG) pathway enrichment analysis of metabolites was performed using a process independently developed by Majorbio Bio-Pharm Technology and enrichment analysis was performed using Fisher’s exact test.

### *In vitro* effects of L-glutamate on skin cells and macrophages

MSFs and immortalized human epidermal (HaCaT) cells were purchased from Shanghai Fuheng Biotechnology Co. Ltd. MSFs and HaCaT cells at passages 2–4 were seeded at 5000–8000 cells/well in 96-well plates for 24 h and cultured with various concentrations of L-glutamate (0, 0.5, 2.5, 5, 10 and 20 μg/ml) in triplicate. Cell survival rate was determined using the CCK-8 assay for 24 h, and the effect of L-glutamate on the proliferation of MSFs and HaCaT cells was analyzed. RAW 264.7 cells were seeded (2 × 10^5^/well) in a 24-well plate and cultured with various concentrations of L-glutamate (0, 5, 10 and 20 μg/ml) for 24 h. Then, the medium was replaced with serum-free medium containing lipopolysaccharide (500 ng/ml; M1 macrophage inducer) or IL-4 (20 ng/ml, M2 macrophage inducer) for 24 h. The control group did not receive any treatments. Finally, the effect of L-glutamate on macrophage polarization of RAW 264.7 cells was evaluated.

A scratch assay was conducted to investigate the effect of L-glutamate on the migration of MSFs. Before plating, the back of the 6-well plate was marked with equally spaced horizontal lines using a marker. MSFs of the logarithmic growth stage were inoculated into the 12-well plate with 1 ml per well and cultured in a constant temperature incubator at 37°C and 5% CO_2_ until the cells formed a monolayer. A scratch on each well was made with a 20-μl micropipette tip, washed thrice with phosphate-buffered saline and mixed with L-glutamate (0, 0.5, 2.5, 5, 10 and 20 μg/ml). Cell migration was observed under a microscope at 0 and 24 h, and the scratch area was analyzed using the ImageJ 2.1.0/1.53c software to assess cell mobility.

### Statistical analysis

All results are expressed as the mean ± standard deviation (SD) of at least three independent experiments. The Shapiro–Wilk test was employed for normality testing. The Student’s *t*-test was used for statistical significance analysis between two groups. One-way analysis of variance was used to analyze significant differences between multiple groups. Two-way analysis of variance was used with two independent variables. The Bonferroni method was performed as a *post hoc* test following analysis of variance. Statistical significance was set at *P* < 0.05. GraphPad Prism 9 (GraphPad Software Inc., La Jolla, CA, USA) was used for all statistical analyses.

## Results

### CGE ameliorates diabetic wound healing

**Figure 1:**
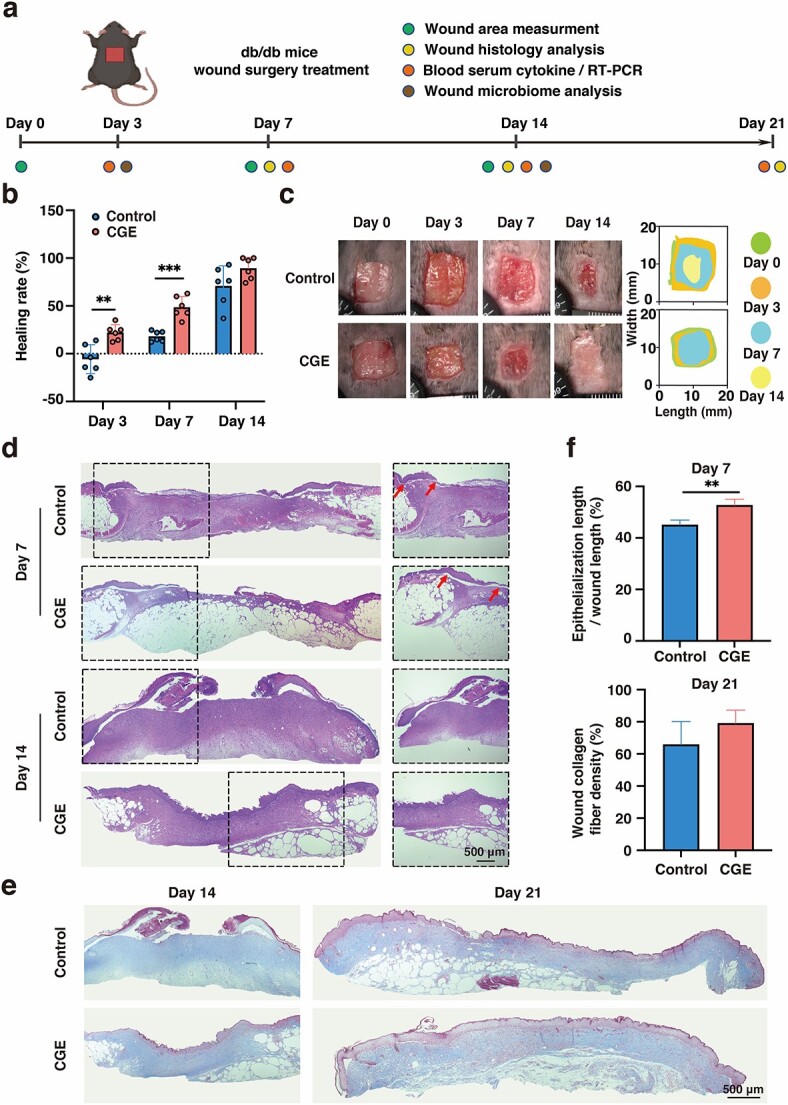
CGE accelerated diabetic wound healing and promoted wound re-epithelialization and collagen fiber deposition. (**a**) Experimental outline; (**b**) wound closure rates of different groups; (**c**) representative images of the healing process in wounds treated with control and CGE; (**d**) H&E staining of full-thickness diabetic wounds on days 7 and 14, arrows indicate newly formed epidermis, **×**50 (Scale bar: 500 µm); (**e**) Masson’s trichrome staining images of full-thickness diabetic wounds on days 14 and 21, **×**50 (Scale bar: 500 µm); (**f**) analysis of epithelialization on day 7 and collagen fiber density on day 21. Results are shown as means ± SD. ^**^p < 0.01 and ^***^p < 0.001 *vs* control group (n = 4–6). * db/db* diabetes mouse, *RT-PCR* reverse transcription-polymerase chain reaction, *CGE calvatia gigantea* extract, *H&E* hematoxylin and eosin, *SD* standard deviation

The optimal concentration of CGE for MSF proliferation was determined *in vitro* and the results are shown in Figure S2 (see online supplementary material). The CGE concentration to treat a db/db mouse wound was set as 100 μg/ml, as previously reported [33]. To investigate the effect of CGE on diabetic wound healing, CGE solution was applied to the diabetic wounds, with normal saline as a blank control. As shown in Figure 1a, diabetic wounds were surgically induced in the db/db mice. Figure 1b and c show the size changes in the diabetic wounds of different groups on days 3, 7 and 14 post-injury. CGE-treated mice exhibited significantly accelerated diabetic wound repair. The wound healing rates were 22.17 ± 3.39, 48.63 ± 4.64 and 89.38 ± 4.66% in the CGE group and −5.80 ± 6.20, 18.33 ± 2.25 and 70.99 ± 8.57% in the control group on days 3, 7 and 14, respectively. On day 3, wounds in the CGE group exhibited a clear wound bed and contraction. By day 14, ~100% of the wounds in the CGE group had healed and the regenerating skin was consistent with the normal skin. In contrast, wounds in the control group did not heal completely, which is consistent with previous reports of delayed healing in db/db mice [34].

### CGE relieves DU-related histological factors

Histological analysis via H&E staining confirmed rapid re-epithelialization in CGE-treated wounds on days 7 and 14 (Figure 1d). The surrounding tissue in the CGE group exhibited flat skin with distinct layers of epidermis and dermis, although no hair follicles or skin appendages were observed. Re-epithelialization in the center of the wound bed was only observed in the CGE group on day 14. H&E staining revealed a severe inflammatory response, with the edges of the wounds filled with granular tissue and no distinctive layers in the control group on days 7 and 14 (Figure 1d). Collagen deposition in the wound area on day 21 was confirmed via Masson’s trichrome staining (Figure 1e). Statistical analysis showed that the epithelialization rate in the CGE group was 52.75 ± 1.14% on day 7, which was significantly higher than that of the control group (45.06 ± 1.09%; Figure 1f). Higher density of collagen fibers was observed in the CGE-treated healed wounds on day 21 than in the open control wounds (Figure 1f).

### CGE regulates the macrophage phenotype

**Figure 2:**
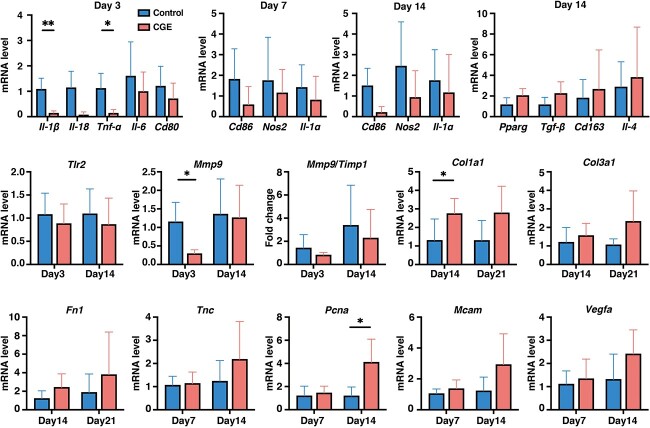
CGE promoted the transition from inflammatory stage to proliferative stage by regulating macrophage phenotype. The mRNA expression levels of *Il-1β*, *Il-18*, *Tnf-α*, *Il-6*, *Cd80*, *Cd86*, *Nos2*, * Il-1α*, *Tlr2*, *Pparγ*, *Tgf-β*, *Cd163*, *Il-4*, *Mmp9*, *Timp1*, *Col1α1, Col3α1*, *Fn1*, *Tnc*, *Pcna*, *Mcam* and *Vegfa* in wound tissues were determined by RT-PCR. Results are shown as means ± SD. ^*^p < 0.05 and ^**^p < 0.01 *vs* control group (n = 3–6). *CGE Calvatia gigantea* extract, *Il-1β,* interleukin 1β, *Tnf-α* tumor necrosis factor α, *Cd80* cluster of differentiation 80, *Nos2* nitric oxide synthase 2, *Tlr2* toll-like receptor 2*, Pparγ* peroxisome proliferator-activated receptor γ*, Tgf-β* transforming growth factor β*, Mmp9* matrix metalloproteinase 9*, Timp1* tissue inhibitor of metalloproteinase 1, *Col1α1* collagen type I α1, * Col3α1* collagen type III α1*, Fn1* fibronectin, *Tnc* tenascin-c, *Pcna* proliferating cell nuclear antigen, *Mcam* melanoma cell adhesion molecule, *Vegfa* vascular endothelial growth factor a, *SD* standard deviation

Transition from the inflammatory stage to the cellular proliferative stage is the key factor in diabetic wound healing. Decrease in the proportion of M1-type pro-inflammatory macrophages and increase in the proportion of M2-type anti-inflammatory macrophages help to regulate the inflammatory response and smoothly transition to the proliferative stage, which is essential for better wound healing, in diabetic wounds. As inflammation is a key factor in diabetic wound healing, we determined the serum levels of inflammatory cytokines (Figure S3, see online supplementary material) and mRNA expression levels of pro-inflammatory factors (*Il-1β*, *Il-18*, *Tnf-α*, *Il-6*, *Cd80*, *Cd86*, *Nos2* and *Il-1α*) and anti-inflammatory factors (*Pparγ*, *Tgf-β*, *Cd163* and *Il-4*) on days 3, 7 and 14 in the tissues (Figure 2). Neither wounding nor CGE treatment affected the systemic inflammatory response (Figure S3, see online supplementary material). In the wound, expression levels of pro-inflammatory genes in the CGE group on day 3 were lower than those in the control group, and expression of *Il-1β*, *Il-18* and *Tnfa* was significantly inhibited (Figure 2). On day 7, expression of proinflammatory genes, including *Cd86*, *Nos2* and *Il-1α*, was suppressed in the CGE group, and a small upward trend was observed in the expression of genes associated with proliferation and angiogenesis, such as *Pcna*, *Mcam*, and *Vegfa*. By day 14, levels of anti-inflammatory genes in the CGE group were higher than those in the control group, and the expression levels of genes related to proliferation, angiogenesis and tissue remodeling continued to increase until day 21 (Figure 2). Immunofluorescence staining revealed that M2 macrophages were upregulated in the wounds after CGE treatment (Figure S4, see online supplementary material). Macrophage polarization accelerates wound healing [35]. Here, markers of M1 macrophages were downregulated, whereas those of M2 macrophages were upregulated, indicating that CGE accelerates diabetic wound healing by regulating macrophage polarization.

### CGE reshapes the wound microbiome in diabetic mice

**Figure 3:**
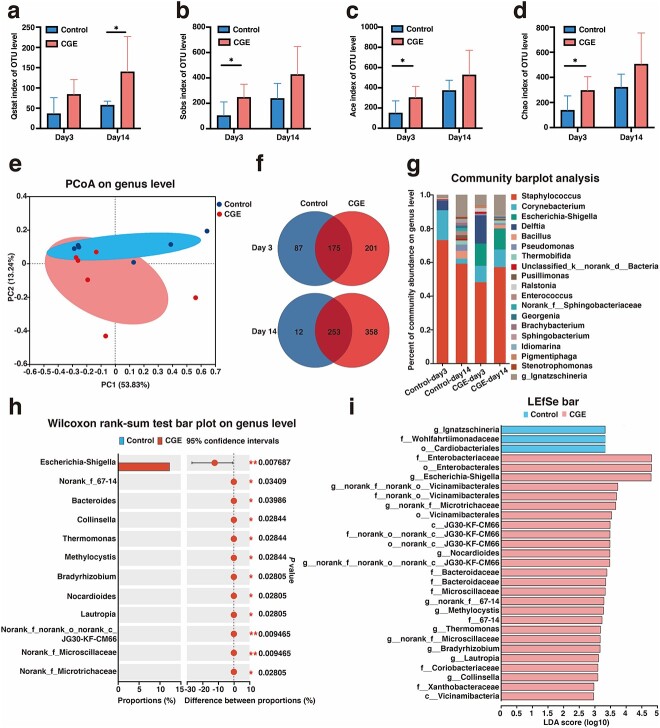
CGE regulated the diabetic wound microbiota based on *16 s rRNA* gene sequencing analysis. α-Diversity indicated by (**a**) the qstat index, (**b**) the sobs index, (**c**) the ace index and (**d**) the chao index. (**e**) PCoA plots used to visualize differences in Soergel distances of samples of OTUs from different groups; (**f**) Venn maps reflected the populations of microorganisms common and unique to different groups at genus level; (**g**) relative abundance plots displayed the differences in the microbial community structure at the genus level; (**h**) extended error bar plot analysis at the genus level analyzed the different microorganisms in the two groups; (**i**) LEfSe analysis analyzed multilevel species differences between the two groups. Results are shown as means ± SD. ^*^P < 0.05 *vs* control group (n = 6). *CGE Calvatia gigantea* extract, *PCoA* principal coordinate analysis, *OTUs* operational taxonomic units, *LEfSe* linear discriminant analysis effect size, *LDA* linear discriminant analysis, *SD* standard deviation

Wound microbiota, formed by the complex colonization of the wound by microorganisms, plays an important role in host health. Wound microbiota has recently been used as a novel target for the diagnosis, prognosis and treatment of tissue repair [36]. In this study, we investigated whether CGE improves wound healing in db/db mice by regulating the wound microbiota via *16S rRNA* gene sequencing analysis. α-Diversity indices were used to evaluate the microbial diversity of different wound groups. The qstat, sobs, ace and Chao indices showed that CGE increased the diversity and abundance of the wound microbiota on days 3 and 14 (Figure 3a–d). PCoA and a Venn diagram revealed a significant difference between the control and CGE groups, with a drastic change in the microbiome diversity of CGE-treated wounds (Figure 3e and f). To further explore the bacteria promoting diabetic wound healing and alleviating inflammation in mice, we analyzed community abundance at the genus level. At the genus level, *Staphylococcus*, *Corynebacterium* and *Escherichia–Shigella* were abundant in both groups. *Staphylococcus* abundance in the CGE group was lower than that in the control group, and *Escherichia–Shigella* were the only bacteria enriched in the CGE group (Figure 3g). Extended error bar plot analysis and LEfSe revealed that *Escherichia–Shigella* proportions were significantly increased in the wounds of CGE-treated mice on day 14, showing the most significant difference. Therefore, *Escherichia–Shigella* were the key genera observed in the diabetic wounds of CGE-treated mice (Figure 3h and i).

**Figure 4:**
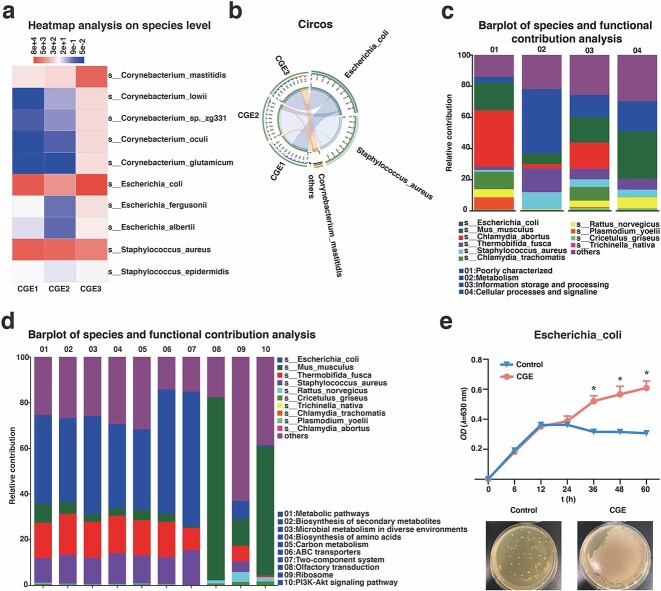
CGE might promote diabetic wound healing by regulating *E. coli* based on metagenomics analysis and *in vitro* bacterial tests. (**a**) Community heatmap shows the 10 species with the highest abundance; (**b**) Circos sample and species relationship map reflects the relationship between the species with the highest abundance and the sample using the RPKM abundance calculation method; (**c**) analysis of species and degree of contribution of COG function; (**d**) analysis of species and degree of contribution of KEGG function; (**e**) dilution plate method and growth curve method were used to verify the proliferative effect of CGE on *E. coli*. Results are shown as means ± SD. ^***^p < 0.001 *vs* control group (n = 6). *CGE Calvatia gigantea* extract*, RPKM* reads per kilobase million, *COG* clusters of orthologous groups of proteins, *KEGG* Kyoto Encyclopedia of genes and genomes, *OD* optical density, *SD* standard deviation, *E. coliEscherichia coli*

To further identify the dominant *Escherichia–Shigella* bacteria discovered above at the species level, we analyzed wound microbiome samples of mice treated with CGE on day 14 using metagenomics analysis. The community heatmap, Circos sample and species relationship map all confirmed that *Staphylococcus aureus*, *E. coli* and *Corynebacterium mastitidis* had the highest abundance in CGE samples, and *E. coli* was the species with the highest proportion in *Escherichia–Shigella* (Figure 4a and b). By analyzing the contribution of species to Clusters of Orthologous Groups of proteins and KEGG functions, *E. coli* had the highest contribution in metabolism. The relative contribution of *E. coli* to metabolic pathways and the biosynthesis of secondary metabolites was the largest (Figure 4c and d). Next, we used the dilution plate and growth curve methods to verify the proliferative effect of CGE on *E. coli*. The effects on *E. coli* of CGE were determined using the dilution plate method. After CGE was applied to *E. coli* for 24 h, they grew rapidly and almost completely covered the surface of the solid medium. The microbial growth curve indicated no difference between the two groups at 24 h post-treatment. However, the OD of the CGE group was higher than that of the control group after 36 h (Figure 4e).

### Effects of CGE on *E. coli* metabolites

**Figure 5:**
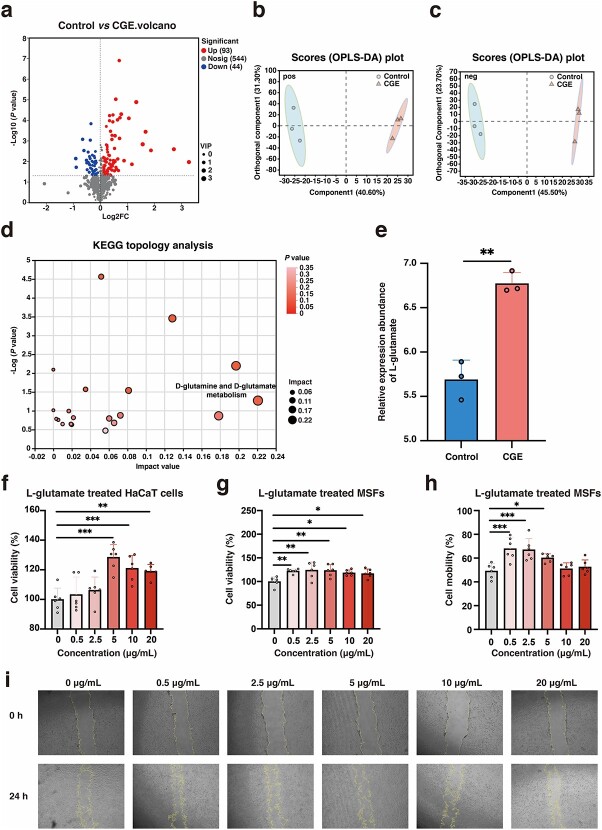
CGE promoted the cell proliferation and migration of skin cells by promoting the secretion of L-glutamate by *E. coli*. (**a**) Volcano plot of the control group and the CGE group metabolites, significant metabolites were selected using the criteria of VIP ≥1 and *p* < 0.05 in the volcano plot. (**b**) OPLS-DA score plot of control group and CGE group at cationic level. (**c**) OPLS-DA score plot of control group and CGE group at anion level. (**d**) KEGG topology analysis was performed for metabolites in the control group and the CGE group. Each bubble represents a KEGG pathway. The horizontal axis represents the magnitude of the impact value of the relative importance of metabolites in the pathway. The vertical axis represents the enrichment significance of metabolite participation pathway −log10 (*p*-value). Bubble size represents impact value. The larger the bubble, the more important the path. (**e**) Histogram of relative expression abundance of medium L-glutamate in different groups of samples. (**f**) *In vitro* cell experiment of L-glutamate promoting HaCaT cell proliferation. (**g**) In vitro cell experiment of L-glutamate promoting MSF proliferation. (**h**) *In vitro* cell experiment of L-glutamate promoting MSF migration. (**i**) Representative images of different concentrations of L-glutamate at 0 and 24 h promoting MSF migration. Results are shown as means ± SD. ^*^p < 0.05, ^**^p < 0.01 and ^***^p < 0.001 *vs* control group (n = 3–6). *CGE calvatia gigantea* extract*, VIP* variable importance in projection, *OPLS-DA* orthogonal partial least squares discriminant analysis, *KEGG* Kyoto Encyclopedia of Genes and Genomes, *HaCaT* immortalized human epidermal, *MSFs* mouse skin fibroblasts, *FC* fold-change, *SD* standard deviation, *E. coliEscherichia coli*

To determine whether CGE promotes healing by regulating *E. coli* in diabetic wounds through a specific metabolite, we used metabolomics to analyze *E. coli* treated with CGE. Volcanic map analysis showed that 93 significantly upregulated metabolites and 44 significantly downregulated metabolites were identified in *E. coli* in the CGE group compared to the control group (Figure 5a). To further analyze the differences between the control group and the CGE group, we used the OPLS-DA model as a supervised technique at the cationic and anion levels (Figure 5b and5c). The validation parameters of fitness (*R*^2^X = 0.719 and *R*^2^Y = 0.995) and predictability (*Q*^2^ = 0.939) of the OPLS-DA model at the cationic level and the validation parameters of fitness (*R*^2^X = 0.693 and *R*^2^Y = 0.998) and predictability (*Q*^2^ = 0.965) of the OPLS-DA model at the anionic level proved that the OPLS-DA diagram had good stability. As shown in Figure 5b and c, the metabolomic characteristics of the control and CGE groups were completely separated, indicating that the metabolites of *E. coli* treated with CGE underwent significant changes. Using the annotated metabolites, we performed metabolite pathway enrichment analysis separately for the control and CGE groups using the KEGG database. This analysis aimed to identify the metabolic pathways and *E. coli* metabolites most influenced by CGE. The significance of each pathway was determined by its impact value, which reflects its relative importance in the context of this study. As shown in Figure 5d, the metabolic pathway most affected by CGE in *E. coli* was D-glutamine and D-glutamate metabolism. This pathway only affected the differential metabolite L-glutamate between the control and CGE groups, and the relative expression of this metabolite was significantly higher in the CGE group than in the control group (Figure 5e).

**Figure 6:**
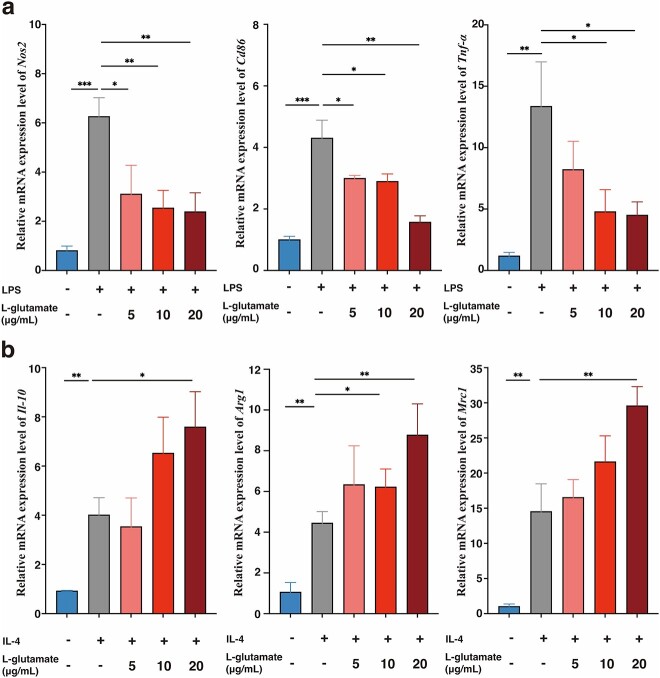
L-Glutamate significantly inhibited M1 macrophage differentiation and promoted M2 macrophage differentiation. mRNA expression levels of (**a**) *Nos2*, *Cd86* and *Tnf-α* and (**b**) *Il-10*, *Arg1* and *Mrc1* were determined by RT-PCR. Results are shown as means ± SD. ^*^p < 0.05, ^**^p < 0.01 and ^***^p < 0.001 *vs* model group (n = 3). *LPS* lipopolysaccharide, *IL-4* interleukin-4, *Nos2* nitric oxide synthase 2, *Cd86* cluster of differentiation 86, *Tnf-α* tumor necrosis factor α, *RT-PCR* reverse transcription-polymerase chain reaction, *Il-10* interleukin 10, *Arg1* arginase 1, *Mrc1* mannose receptor C-type 1, *SD* standard deviation

To further clarify whether L-glutamate produced by *E. coli* after CGE treatment is a key factor promoting wound healing, we examined the effects of L-glutamate on the proliferation and migration of MSFs and HaCaT cells. As shown in Figure 5f–i, L-glutamate significantly promoted the proliferation of HaCaT cells at 5–20 μg/ml and MSFs at 0.5–20 μg/ml. Moreover, L-glutamate significantly promoted the migration of MSFs at 0.5–5 μg/ml. Effects of L-glutamate on macrophage differentiation were also investigated. L-Glutamate significantly inhibited lipopolysaccharide-induced M1 polarization at 5–20 μg/ml (Figure 6a) and promoted M2 polarization at 20 μg/ml (Figure 6b). Therefore, changes in L-glutamate were important in mediating the inflammatory response and macrophage differentiation during healing in the CGE group.

## Discussion

DUs are a leading cause of mortality and mobility in individuals with diabetes. DUs are mainly treated via surgery and adjuvant therapies. However, no gold standard treatment is currently available for diabetic wound healing [37–39]. Here, we selected CG for the treatment of DUs owing to its use in traditional Chinese medicine and modern clinical applications [27]. We found that CGE regulated the wound microbiota and promoted macrophage II polarization, leading to a faster transition from the inflammatory stage to the proliferative stage, enhanced epithelialization and better matrix remodeling. Our findings suggest the commensal wound microbiome as a potential target for DU treatment.

Skin microbiome plays an important role in wound healing [40]. Over 90% of the bacteria in DUs are pathogenic bacteria, among which *Staphylococcus aureus* is the most prevalent species [41]. In this study, microbial diversity and richness in wounds were significantly improved after CGE treatment. A diverse microbiome provides health benefits by resisting pathogen colonization [42]. Patients with DUs exhibit a lower diversity of the wound microbiome than healthy individuals, which affects their healing process [43]. Here, imbalance in the DU wound microbiome was restored by CGE treatment, as revealed via *16S rRNA* gene sequencing analysis. For the first time, our results revealed *E. coli* as the most significantly enriched species in CGE-treated wounds, suggesting it as the key commensal bacterium promoting diabetic wound healing. *E. coli* is a normal resident bacterium in the intestines of animals as well as a skin symbiotic bacterium [44]. Most *E. coli* bacteria exist in a symbiotic relationship with humans, with some species even used as probiotics. Nissl 1917 is a typical probiotic species of *E. coli* [45] that mainly strengthens the mucosal barrier, regulates the secretion of immune factors, inhibits pathogens and toxins, and maintains bacterial homeostasis [46]. Here, metabolomic analysis revealed that D-glutamine and D-glutamate metabolism had the highest impact on the KEGG pathway. This metabolic pathway was also associated with the differential metabolite, L-glutamate, in the control and CGE groups. Recent studies have shown a positive correlation among the symbiotic wound bacterial load, glutamine metabolism and regeneration. Symbiotic skin bacteria accelerate skin and hair follicle regeneration by increasing glutamine metabolism in keratinocytes [47]. Here, L-glutamate promoted the proliferation of HaCaT cells, migration of MSFs and macrophage polarization, contributing to epithelialization and tissue barrier regeneration in diabetic wounds.

Macrophages are key players in inflammation [48]. As the inflammatory phase progresses [49], ‘pro-inflammatory’ M1 macrophages skew toward the ‘pro-healing’ M2 phenotype [50]. M1 macrophages are responsible for the phagocytosis of bacteria and tissue debris and the release of pro-inflammatory cytokines [51]. M2 macrophages exert anti-inflammatory and immunomodulatory effects and promote wound healing [52]. During the inflammatory stage of diabetic wound healing, CGE treatment downregulated the expression levels of M1-polarized marker genes (*Il-1β*, *Tnf-α*, *Il-6*, *Cd86* and *Nos2*) and upregulated the expression levels of M2-polarized marker genes (*Il-4*, *Tnf-α* and *Cd163*). These changes in macrophage polarization may be attributed to the balanced skin microbiota after CGE treatment that interacts with various immune cells involved in wound healing [53]. Similarly, engineered *Lactococcus lactis* accelerates diabetic wound healing by promoting angiogenesis and macrophage polarization [34]. Therefore, CGE improves the wound microenvironment by promoting M1-to-M2 macrophage transformation. M2 macrophages suppress inflammation and promote angiogenesis. In addition, M2 macrophages activate fibroblasts to differentiate into myofibroblasts, thereby secreting collagen and promoting tissue repair [54]. Consistent with previous reports, we also observed an increase in the gene expression levels of *Vegfa*, *Col1α1* and *Col3α1* related to angiogenesis and tissue regeneration in diabetic wounds in this study.

## Conclusions

In summary, our investigation revealed that CGE accelerated the healing of DUs by modulating the wound microbiota. Mechanistically, CGE increased the diversity of the wound microbiome, mitigated the abundance of pathogenic bacteria, such as *Staphylococcus aureus*, and notably enriched *E. coli*. Consequently, CGE treatment facilitated a more balanced wound microbiota and further promoted the polarization of macrophages towards the M2 phenotype. The stimulation of M2 polarization was attributed to the secretion of L-glutamate by *E.coli*, which facilitated the proliferation and migration of keratinocytes and fibroblasts, thereby enhancing epithelialization and tissue barrier regeneration. Future studies will delve into the correlation between DU microbiota and wound immune responses to determine whether macrophage phenotype transformation is closely related to changes in the wound microbiota composition. In conclusion, our findings underscore CGE as a potential therapeutic intervention for DUs.

## Abbreviations

*Actβ*: Actin β; *Arg1*: Arginase 1; CCK: Cell counting kit; *Cd163*: Cluster of differentiation 163; CG: *Calvatia gigantea*; CGE: *Calvatia gigantea* extract; *Col1α1*: Collagen type I α1; *Col3α1*: Collagen type III α1; DAPI: 4′,6-Diamidino-2-phenylindole; DUs: Diabetic ulcers; *Fn1*: Fibronectin; H&E: Hematoxylin and eosin; HaCaT: Immortalized human epidermal; IFN-γ: Interferon-γ; IL-10 Interleukin-10; *Il-18*: Interleukin 18; KEGG: Kyoto encyclopedia of genes and genomes; LB: Luria–Bertani; LEfSe: Linear discriminant analysis effect size; *m*/*z*: Mass to charge ratio; *Mcam*: Melanoma cell adhesion molecule; *Mmp9*: Matrix metalloproteinase 9; *Mrc1*: Mannose receptor C-type 1; MS/MS: Tandem mass spectrometry; MSFs: Mouse skin fibroblasts; *Nos2*: Nitric oxide synthase 2; OD: Optical density; OPLS-DA: Orthogonal partial least squares discriminant analysis; *Pcna*: Proliferating cell nuclear antigen; PCoA: Principal coordinate analysis; *Pparγ*: Peroxisome proliferator-activated receptor γ; ppm: Part per million; RT-PCR: Reverse transcription-polymerase chain reaction; SD: Standard deviation; *Tgf-β*: Transforming growth factor β; *Timp1*: Tissue inhibitor of metalloproteinase 1; *Tlr2*: Toll-like receptor 2; *Tnc*: Tenascin-c; TNF-α: Tumor necrosis factor-α; HPLC-Q-TOF-MS/MS: Ultra-high performance liquid chromatography-quadrupole time-of-flight tandem mass spectrometry; *Vegfa*: Vascular endothelial growth factor a.

## Supplementary Material

Supplementary_materials_tkae037
