# Supplementary material for: Reshaped commensal wound microbiome via topical application of Calvatia gigantea extract contributes to faster diabetic wound healing
Source: Burns Trauma. 2024 Sep 2;12:tkae037. doi: 10.1093/burnst/tkae037 (PMC11367672; doi:10.1093/burnst/tkae037)
Supplement: Supplementary_materials_tkae037 [file supplementary_materials_tkae037.docx]

**Supplementary material of the manuscript****:** **Reshaped commensal wound microbiome via topical application of *Calvatia gigantea* extract contributes to faster diabetic wound healing**

**Supplementary Figures**

**
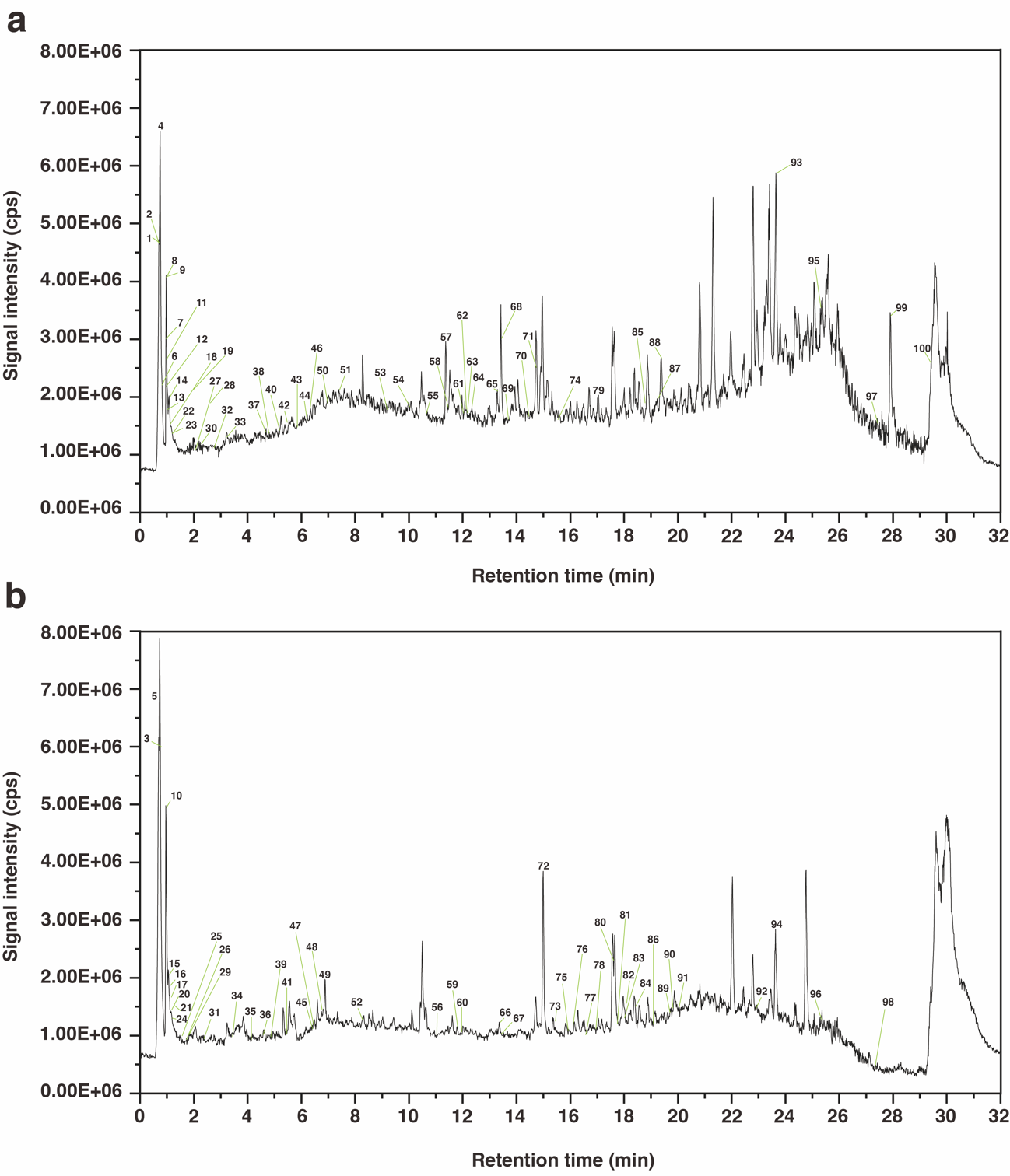
**

**Figure S1****.** UHPLC-Q-TOF-MS/MS analysis of CGE compounds

(a) Total ion chromatogram of CGE components (positive ionization); (b) Total ion chromatogram of CGE components (negative ionization). The numbers in the figure represent the serial numbers of compounds in Table S1. *UHPLC-Q-TOF-MS/MS* ultra-high performance liquid chromatography-quadrupole time-of-flight tandem mass spectrometry, *CGE calvatia gigantea* extract

**Table S1. List of** **CGE compound identification information**

| **No.** | **t_R_/min** | **Characterization** | **Molecular formula** | **positive ion /** | **m/z** | | **Fragment ion**  **MS/MS** | **ppm** | **Peak area** |
| --- | --- | --- | --- | --- | --- | --- | --- | --- | --- |
|  |  |  |  | **negative ion** | **theoretical** | **measured** |  |  |  |
| 1 | 0.695 | Carnitine | C_7_H_15_NO_3_ | [M+H]^+^ | 162.113 | 162.112 | 60.082;85.030;102.094 103.041;162.112 | 3.89 | 336426 |
| 2 | 0.695 | Gentiobiose | C_12_H_22_O_11_ | [M+NH_4_]^+^ | 360.151 | 360.150 | 85.028;91.038;127.038 145.049;163.060 | 4.39 | 167973 |
| 3 | 0.719 | Gluconic acid | C_6_H_12_O_7_ | [M-H]^-^ | 195.050 | 195.050 | 59.012;71.012;75.007 85.028;87.007 | 1.18 | 1330142 |
| 4 | 0.747 | 4-Guanidinobutyric acid | C_5_H_11_N_3_O_2_ | [M+H]^+^ | 146.092 | 146.093 | 56.054;60.061;69.039 86.065;87.048;111.061 146.099 | -2.67 | 267103 |
| 5 | 0.758 | (s)-Malate | C_4_H_6_O_5_ | [M-H]^-^ | 133.015 | 133.014 | 71.012;115.001;133.012 | 10.45 | 871197 |
| 6 | 0.813 | Nicotinic acid | C_6_H_5_NO_2_ | [M+H]^+^ | 124.039 | 124.039 | 80.048;96.044;124.039 | 3.15 | 199709 |
| 7 | 0.959 | Uracil | C_4_H_4_N_2_O_2_ | [M+H]^+^ | 113.034 | 113.034 | 70.029;96.008;113.039 | -4.87 | 49384 |
| 8 | 0.973 | Hypoxanthine | C_5_H_4_N_4_O | [M+H]^+^ | 137.045 | 137.046 | 55.029;82.040;94.041 110.035;119.034;137.046 | -6.42 | 70715 |
| 9 | 0.973 | Adenosine | C_10_H_13_N_5_O_4_ | [M+H]^+^ | 268.103 | 268.103 | 119.036;136.617;268.109 | -0.41 | 213692 |
| 10 | 0.976 | N-Fructosyl pyroglutamate | C_11_H_17_NO_8_ | [M-H]^-^ | 290.089 | 290.087 | 201.830 | 9.00 | 129623 |
| 11 | 1.000 | N-Acetylglutamic acid | C_7_H_11_NO_5_ | [M+H]^+^ | 190.070 | 190.071 | 84.043;102.055;148.062 | -4.21 | 39042 |
| 12 | 1.013 | Tyrosine | C_18_H_32_O_7_ | [M+H]^+^ | 182.082 | 182.081 | 77.040;91.054;95.049 119.052;123.045;136.074 | 1.65 | 16103 |
| 13 | 1.039 | Guanosine | C_10_H_13_N_5_O_5_ | [M+H]^+^ | 284.099 | 284.098 | 135.029;152.055 | 1.62 | 19592 |
| 14 | 1.065 | Inosine | C_10_H_12_N_4_O_5_ | [M+H]^+^ | 269.088 | 269.088 | 94.039;110.035;119.034 137.046;137.061 | 1.34 | 27575 |
| 15 | 1.067 | Inosine | C_10_H_12_N_4_O_5_ | [M-H]^-^ | 267.075 | 267.073 | 135.030;267.073 | 6.78 | 42359 |
| 16 | 1.092 | Succinic acid | C_4_H_6_O_4_ | [M-H]^-^ | 117.021 | 117.018 | 73.029;117.017 | 24.79 | 1016345 |
| 17 | 1.105 | Citramalate | C_5_H_8_O_5_ | [M-H]^-^ | 147.030 | 147.028 | 57.037;85.032;87.010 | 12.72 | 41212 |
| 18 | 1.145 | Thymine | C_5_H_6_N_2_O_2_ | [M+H]^+^ | 127.050 | 127.050 | 82.028;84.044;109.039 110.023;127.050 | -0.08 | 45147 |
| 19 | 1.145 | 4-Quinolinecarboxylic acid | C_10_H_7_NO_2_ | [M+H]^+^ | 174.055 | 174.055 | 128.051;130.067;146.061 174.056 | -1.09 | 14324 |
| 20 | 1.155 | 3-Hydroxy-3-methylglutaric acid | C_6_H_10_O_5_ | [M-H]^-^ | 161.046 | 161.044 | 57.036;59.017;99.040  101.022;161.044 | 14.29 | 59777 |
| 21 | 1.155 | 4-Pyridoxic acid | C_8_H_9_NO_4_ | [M-H]^-^ | 182.045 | 182.046 | 108.047;138.055;138.073 182.042 | -6.10 | 43629 |
| 22 | 1.172 | 6-Hydroxynicotinic acid | C_6_H_5_NO_3_ | [M+H]^+^ | 140.035 | 140.034 | 51.022;66.034;78.033 94.028;122.022;140.033 | 1.86 | 152970 |
| 23 | 1.172 | 4-Acetamidobutyric acid | C_6_H_11_NO_3_ | [M+H]^+^ | 146.080 | 146.080 | 69.035;86.060;87.044 | 0.68 | 23059 |
| 24 | 1.180 | 6-Hydroxynicotinate | C_6_H_5_NO_3_ | [M-H]^-^ | 138.022 | 138.019 | 94.029;94.041 | 21.74 | 124780 |
| 25 | 1.532 | 3'-O-Methylinosine | C_11_H_14_N_4_O_5_ | [M-H]^-^ | 281.090 | 281.088 | 135.029;281.084 | 7.12 | 22632 |
| 26 | 2.035 | Glutaric acid | C_5_H_8_O_4_ | [M-H]^-^ | 131.036 | 131.034 | 69.037;87.045 | 10.76 | 121730 |
| 27 | 2.049 | p-Aminobenzoic acid | C_7_H_7_NO_2_ | [M+H]^+^ | 138.054 | 138.054 | 77.040;77.052;94.069 94.072;138.055 | -4.78 | 45312 |
| 28 | 2.049 | Pantothenic acid | C_9_H_17_NO_5_ | [M+H]^+^ | 220.117 | 220.118 | 90.054;116.034;184.094 202.107;220.116 | -0.45 | 300652 |
| 29 | 2.073 | Pantothenate | C_9_H_17_NO_5_ | [M-H]^-^ | 218.103 | 218.103 | 71.016;71.052;88.039 99.049;116.076;146.085 218.105 | 0.96 | 477355 |
| 30 | 2.232 | Acetaminophen | C_8_H_9_NO_2_ | [M+H]^+^ | 152.071 | 152.070 | 65.038;92.049;93.033 110.060;152.069 | 7.24 | 383853 |
| 31 | 2.264 | Paracetamol | C_8_H_9_NO_2_ | [M-H]^-^ | 150.054 | 150.056 | 107.037;150.055 | -8.33 | 24095 |
| 32 | 2.789 | 4-Hydroxyquinoline | C_9_H_7_NO | [M+H]^+^ | 146.060 | 146.060 | 77.037;77.041;91.056 117.051;128.047;146.061 | 1.10 | 134907 |
| 33 | 3.196 | Kynurenic acid | C_10_H_7_NO_3_ | [M+H]^+^ | 190.050 | 190.050 | 89.041;116.052;144.045 162.058;190.049 | 1.16 | 82398 |
| 34 | 3.453 | 2-Isopropylmalic acid | C_7_H_12_O_5_ | [M-H]^-^ | 175.062 | 175.061 | 93.088 | 8.57 | 88220 |
| 35 | 4.222 | 2,3-Dihydroxybenzoic acid | C_7_H_6_O_4_ | [M-H]^-^ | 153.020 | 153.020 | 65.006;91.022 | -1.96 | 56413 |
| 36 | 4.492 | 2-Hydroxy-4-methylpentanoic acid | C_6_H_12_O_3_ | [M-H]^-^ | 131.071 | 131.071 | 85.065;131.070 | 3.28 | 127530 |
| 37 | 4.647 | 2-Hydroxybenzonitrile | C_7_H_5_NO | [M+H]^+^ | 120.044 | 120.044 | 65.038;92.049;120.044 | 4.17 | 52892 |
| 38 | 4.903 | Deoxyvasicinone | C_11_H_10_N_2_O | [M+H]^+^ | 187.087 | 187.086 | 77.040;89.031;92.053 141.054;187.086 | 2.73 | 31478 |
| 39 | 4.951 | Paeoniflorin | C_23_H_28_O_11_ | [M+HCOO]^-^ | 525.155 | 525.161 | 121.026;327.103;449.136 449.148 | ###### | 20123 |
| 40 | 5.173 | Cyclo(Leu-Pro) | C_11_H_18_N_2_O_2_ | [M+H]^+^ | 211.144 | 211.143 | 70.065;86.097;98.060 211.143 | 1.37 | 35801 |
| 41 | 5.436 | 3-Phenyllactic acid | C_9_H_10_O_3_ | [M-H]^-^ | 165.055 | 165.055 | 72.994;101.040;103.053 119.049;147.043 | -2.42 | 152751 |
| 42 | 5.573 | N-Acetylphenylalanine | C_11_H_13_NO_3_ | [M+H]^+^ | 208.097 | 208.096 | 77.038;79.053;91.054 103.054;120.080 | 3.27 | 11763 |
| 43 | 5.853 | Pyrrolo[1,2-a]pyrazine-1,4-dione, hexahydro-3-(phenylmethyl)- | C_14_H_16_N_2_O_2_ | [M+H]^+^ | 245.129 | 245.128 | 70.064;120.080;154.074 172.112;217.134 | 5.27 | 33642 |
| 44 | 6.204 | 1H-indole-3-carboxylic acid | C_9_H_7_NO_2_ | [M+H]^+^ | 162.055 | 162.054 | 116.048;117.054;118.064 144.043;162.056 | 4.63 | 39684 |
| 45 | 6.338 | Indole-3-carboxylic acid | C_9_H_7_NO_2_ | [M-H]^-^ | 160.040 | 160.040 | 115.697;116.054;160.039 | 1.81 | 113645 |
| 46 | 6.352 | 3-Formylindole | C_9_H_7_NO | [M+H]^+^ | 146.058 | 146.060 | 91.054;118.065;146.060 | -7.81 | 49135 |
| 47 | 6.478 | Indole-3-carboxaldehyde | C_9_H_7_NO | [M-H]^-^ | 144.047 | 144.046 | 115.044;116.051;126.034 142.033;144.053 | 6.25 | 180552 |
| 48 | 6.730 | 7,8-dimethylalloxazine (lumichrome) | C_12_H_10_N_4_O_2_ | [M-H]^-^ | 241.073 | 241.073 | 198.065;198.067;198.069 241.071;241.075 | 2.07 | 27621 |
| 49 | 6.906 | Azelaic acid | C_9_H_16_O_4_ | [M-H]^-^ | 187.097 | 187.097 | 97.064;123.080;125.096 126.097;187.096 | -1.50 | 857431 |
| 50 | 6.947 | Indole-3-acetic acid | C_10_H_9_NO_2_ | [M+H]^+^ | 176.069 | 176.070 | 130.064;131.063;176.066 | -8.52 | 3548 |
| 51 | 7.392 | N-Methylanthranilic acid | C_8_H_9_NO_2_ | [M+H]^+^ | 152.069 | 152.070 | 106.065;134.059;152.069 | -4.61 | 20597 |
| 52 | 8.301 | Decanedioic acid | C_10_H_18_O_4_ | [M-H]^-^ | 201.113 | 201.113 | 57.037;80.025;111.081 137.094;183.102 | 1.49 | 94453 |
| 53 | 9.251 | N-(1-hydroxy-3-phenylpropan-2-yl)benzamide | C_16_H_17_NO_2_ | [M+H]^+^ | 256.134 | 256.133 | 91.054;105.035;117.070 238.123 | 6.25 | 65195 |
| 54 | 9.987 | (3S,6Z)-3-methyl-6-[[2-(2-methylbut-3-en-2-yl)-1H-indol-3-yl]methylidene]piperazine-2,5-dione | C_19_H_21_N_3_O_2_ | [M+H]^+^ | 324.171 | 324.170 | 155.061;156.067;158.081 169.076;183.091;212.076 239.084;256.108 | 3.06 | 82177 |
| 55 | 10.652 | Paeonol | C_9_H_10_O_3_ | [M+H]^+^ | 167.071 | 167.070 | 51.025;121.066;167.065 167.070 | 4.37 | 41314 |
| 56 | 10.978 | Dodecanedioic acid | C_12_H_22_O_4_ | [M-H]^-^ | 229.143 | 229.144 | 167.144;211.133;229.141 | -3.06 | 14764 |
| 57 | 11.371 | Lauryldiethanolamine | C_16_H_35_NO_2_ | [M+H]^+^ | 274.275 | 274.274 | 57.069;70.065;71.085 88.075;102.091;106.086 256.264 | 3.61 | 3382661 |
| 58 | 11.460 | N,N-Dimethyldodecylamine N-oxide | C_14_H_31_NO | [M+H]^+^ | 230.248 | 230.247 | 57.070;58.065;62.060 71.085;212.236;230.247 | 4.78 | 193913 |
| 59 | 11.818 | FA 18:1+3O | C_18_H_34_O_5_ | [M-H]^-^ | 329.233 | 329.231 | 99.080;169.125;199.131 211.131 | 6.99 | 285615 |
| 60 | 12.011 | Tridecanedioic acid | C_13_H_24_O_4_ | [M-H]^-^ | 243.160 | 243.160 | 179.146;181.158;225.148 243.160 | 2.06 | 37596 |
| 61 | 12.050 | Benzyldiphenylphosphine oxide | C_19_H_17_OP | [M+H]^+^ | 293.110 | 293.109 | 91.054;201.046;293.108 | 2.42 | 160669 |
| 62 | 12.104 | Dicyclohexylurea | C_13_H_24_N_2_O | [M+H]^+^ | 225.195 | 225.196 | 83.085;100.111;143.117 225.194 | -0.93 | 388647 |
| 63 | 12.144 | Biliverdin | C_33_H_34_N_4_O_6_ | [M+H]^+^ | 583.254 | 583.254 | 297.123;299.140;583.253 | 0.14 | 11976 |
| 64 | 12.320 | Aurantiamide | C_25_H_26_N_2_O_3_ | [M+H]^+^ | 403.202 | 403.201 | 105.033;117.069;134.095 152.107;252.101;385.192 | 3.70 | 147212 |
| 65 | 13.287 | 2,2'-(Tetradecylimino)diethanol | C_18_H_39_NO_2_ | [M+H]^+^ | 302.307 | 302.305 | 57.069;70.065;88.075 106.085;284.295;302.305 | 5.99 | 1536125 |
| 66 | 13.385 | FA 18:3+2O | C_18_H_30_O_4_ | [M-H]^-^ | 309.206 | 309.205 | 99.079;185.118;209.114 291.193;309.207 | 2.27 | 150904 |
| 67 | 13.423 | 6:2 fluorotelomer sulfonic acid | C_8_H_5_F_13_O_3_S | [M-H]^-^ | 426.971 | 426.967 | 406.959;406.970;426.965 | 8.92 | 20256 |
| 68 | 13.429 | Phytosphingosine | C_18_H_39_NO_3_ | [M+H]^+^ | 318.300 | 318.300 | 60.045;282.279;300.290 318.319 | 1.92 | 3953475 |
| 69 | 13.736 | Tris(1-chloro-2-propyl)phosphate | C_9_H_18_Cl_3_O_4_P | [M+H]^+^ | 327.005 | 327.008 | 174.990;251.001;252.991 | -6.70 | 22677 |
| 70 | 14.444 | Dihydrosphingosine | C_18_H_39_NO_2_ | [M+H]^+^ | 302.306 | 302.305 | 60.044;254.280;284.294 302.307 | 5.60 | 56322 |
| 71 | 14.739 | 7b,9-Dihydroxy-3-(hydroxymethyl)-1,1,6,8-tetramethyl-5-oxo-1,1a,1b,4,4a,5,7a,7b,8,9-decahydro-9aH-cyclopropa[3,4]benzo[1,2-e]azulen-9a-yl acetate | C_22_H_30_O_6_ | [M+ACN+H]^+^ | 432.238 | 432.238 | 107.084;119.085;135.080 147.067;281.140 | 0.44 | 518431 |
| 72 | 15.012 | 9,10-DiHOME | C_18_H_34_O_4_ | [M-H]^-^ | 313.238 | 313.238 | 171.130;201.112;277.195 295.186;313.171 | 1.18 | 5680318 |
| 73 | 15.390 | LPE 16:1 | C_21_H_42_NO_7_P | [M-H]^-^ | 450.261 | 450.262 | 196.037;214.053;253.215 450.263 | -2.40 | 26167 |
| 74 | 15.664 | Osthol | C_15_H_16_O_3_ | [M+H]^+^ | 245.120 | 245.120 | 77.037;102.047;103.055 131.050;245.116 | 0.41 | 21803 |
| 75 | 15.916 | LPC 18:2 | C_26_H_50_NO_7_P | [M+FA-H]^-^ | 564.333 | 564.330 | 197.185 | 5.78 | 14610 |
| 76 | 16.173 | LPE 18:2 | C_23_H_44_NO_7_P | [M-H]^-^ | 476.278 | 476.277 | 196.037;196.039;214.047 279.232;476.278 | 0.82 | 378853 |
| 77 | 16.604 | LPE 17:1 | C_22_H_44_NO_7_P | [M-H]^-^ | 464.275 | 464.277 | 196.036;196.038;214.049 267.232;464.278 | -5.84 | 23029 |
| 78 | 17.014 | LPE 16:0 | C_21_H_44_NO_7_P | [M-H]^-^ | 452.277 | 452.277 | 196.037;196.039;214.043 255.231;452.277 | -0.02 | 32902 |
| 79 | 17.044 | Asperphenamate [(2S)-2-benzamido-3-phenylpropyl] (2S)-2-benzamido-3-phenylpropanoate | C_32_H_30_N_2_O_4_ | [M+H]^+^ | 507.227 | 507.226 | 105.033;224.106;238.123 239.125;256.133 | 0.41 | 598767 |
| 80 | 17.602 | 12,13-EODE | C_18_H_32_O_3_ | [M-H]^-^ | 295.227 | 295.227 | 195.146;277.211 | -1.93 | 3947248 |
| 81 | 17.940 | LPI 18:2 | C_27_H_49_O_12_P | [M-H]^-^ | 595.288 | 595.288 | 241.011;279.232;315.048 595.286 | -1.48 | 26587 |
| 82 | 18.028 | 9-HODE | C_18_H_32_O_3_ | [M-H]^-^ | 295.229 | 295.227 | 171.102;295.227;277.216 | 4.37 | 430047 |
| 83 | 18.079 | LPI 15:0 | C_24_H_47_O_12_P | [M-H]^-^ | 557.272 | 557.273 | 241.018;241.217;315.049 557.273 | -2.06 | 15990 |
| 84 | 18.436 | Octadecanedioic acid | C_18_H_34_O_4_ | [M-H]^-^ | 313.237 | 313.238 | 251.237;295.227;313.238 | -2.52 | 10072 |
| 85 | 18.816 | Rakicidin B | C_29_H_39_N_3_O_2_ | [M+H]^+^ | 462.312 | 462.312 | 130.065;198.129;210.129 264.176;278.192;338.189 406.252;462.312 | -0.41 | 173889 |
| 86 | 19.054 | LPG 15:0 | C_21_H_43_O_9_P | [M-H]^-^ | 469.254 | 469.257 | 152.992;227.031;241.217 469.257 | -5.54 | 35359 |
| 87 | 19.215 | Di-n-butyl phthalate | C_16_H_22_O_4_ | [M+H]^+^ | 279.161 | 279.159 | 53.007;65.038;93.033 121.040;149.023 | 9.00 | 609189 |
| 88 | 19.374 | Phthalic acid | C_8_H_6_O_4_ | [M-H_2_O+H]^+^ | 149.022 | 149.023 |  | -7.58 | 270163 |
| 89 | 19.710 | 2,6-Di-tert-butyl-4-nitrophenol | C_14_H_21_NO_3_ | [M-H]^-^ | 250.145 | 250.144 | 148.055;205.123;220.149 234.112;250.145 | 2.00 | 68192 |
| 90 | 19.773 | lauryl sulfate | C_12_H_26_O_4_S | [M-H]^-^ | 265.149 | 265.147 | 79.957;96.958;265.149 | 4.19 | 415711 |
| 91 | 20.012 | dl-Norgestrel | C_21_H_28_O_2_ | [M-H]^-^ | 311.201 | 311.201 | 133.065;149.097 | -0.93 | 91333 |
| 92 | 22.942 | Oleyl sarcosine | C_21_H_39_NO_3_ | [M-H]^-^ | 352.284 | 352.285 | 125.095;139.113;141.128 199.228;207.595 | -2.59 | 138135 |
| 93 | 23.645 | Linoleic acid | C_18_H_32_O_2_ | [M+H]^+^ | 281.248 | 281.247 | 69.070;81.069;83.085 95.084;97.100;109.101 111.116;123.116;133.101 | 4.98 | 2234411 |
| 94 | 23.650 | Linoleic acid | C_18_H_32_O_2_ | [M-H]^-^ | 279.232 | 279.232 | 58.010;59.016;71.015 95.047;113.066 | -1.22 | 2676332 |
| 95 | 25.342 | (1S,2R,5R,6R,10R,13S,15S)-5-[(E,2R,5R)-5,6-dimethylhept-3-en-2-yl]-6,10-dimethyl-16,17-dioxapentacyclo[13.2.2.01,9.02,6.010,15]nonadec-18-en-13-ol | C_28_H_44_O_3_ | [M-H_2_O+H]^+^ | 411.325 | 411.325 | 145.101;158.072;173.095 175.111;191.106;309.222 411.327 | 0.95 | 576297 |
| 96 | 25.371 | Trans-Vaccenic acid | C_18_H_34_O_2_ | [M-H]^-^ | 281.248 | 281.248 | 263.236;281.247 | 0.36 | 591256 |
| 97 | 27.372 | Dioctyl Phthalate | C_24_H_38_O_4_ | [M+H]^+^ | 391.283 | 391.284 | 57.070;71.085;149.023 167.033 | -2.05 | 126588 |
| 98 | 27.387 | Tetradecylsulfate | C_14_H_30_O_4_S | [M-H]^-^ | 293.179 | 293.179 | 96.959;293.177 | 1.71 | 159159 |
| 99 | 27.910 | Erucamide | C_22_H_43_NO | [M+H]^+^ | 338.342 | 338.342 | 57.068;71.084;81.069 83.084;85.100;91.052 95.086 | -0.92 | 6215102 |
| 100 | 29.411 | DAG 36:2 | C_39_H_72_O_5_ | [M+NH_4_]^+^ | 638.570 | 638.572 | 339.289;603.559;638.571 | -2.87 | 30706 |

*CGE Calvatia gigantea* extract,*No.* number, *t_R_* retention time, *m/z* mass to charge ratio, *MS/MS* tandem mass spectrometry, *ppm* part per million

**
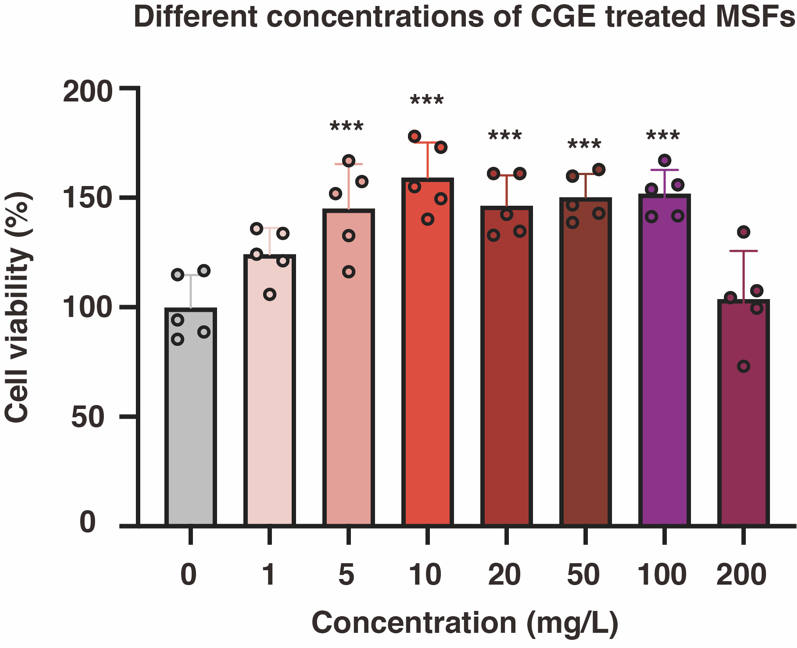
**

**Figure S2.** Screening of CGE concentration for the treatment of db/db mice

*In vitro* cell experiment of promoting MSFs proliferation with different concentrations of CGE. Results are shown as means ± SD. ^**^*p* < 0.001 *vs* control group (concentration: 0 μg/mL) (n=5). *CGE calvatia gigantea* extract, *MSFs* mouse skin fibroblasts, *SD* standard deviation


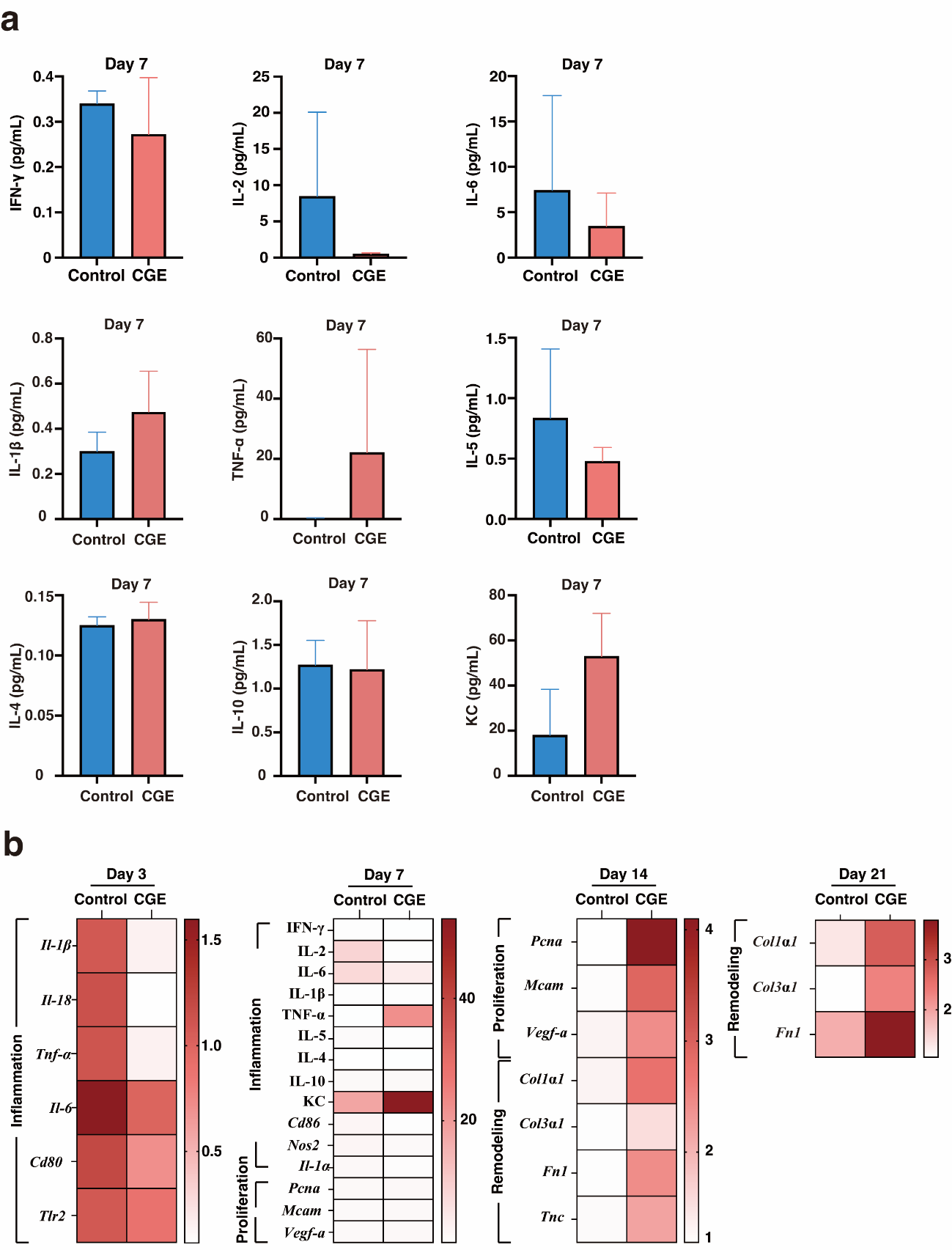


**Figure S3.** Measurement of inflammatory cytokines in serum of db/db mice

(a) Effect of CGE on the serum level of IFN-γ , IL-2, IL-6 , IL-1β, TNF-α, IL-5, IL-4, IL-10 and KC in db/db mice. (b) Key genes representing inflammation, proliferation and remodeling were expressed in different groups on day 3,7,14,21. Results are shown as means ± SD (n=2-6). *CGE calvatia gigantea* extract, *IFN-γ* interferon-γ, *IL* interleukin, *TNF-α* tumor necrosis factor-α, *KC* keratinocyte-derived cytokine, *Cd80* cluster of differentiation 80*, Cd86* cluster of differentiation 86*, Nos2* nitric oxide synthase 2*, Tlr2* toll-like receptor 2, *Col1*α*1* collagen type I α1*, Col3*α*1* collagen type III α1*, Fn1* fibronectin*, Tnc* tenascin-c*, Pcna* proliferating cell nuclear antigen*, Mcam* melanoma cell adhesion molecule*,* *Vegfa* vascular endothelial growth factor a, *SD* standard deviation

**
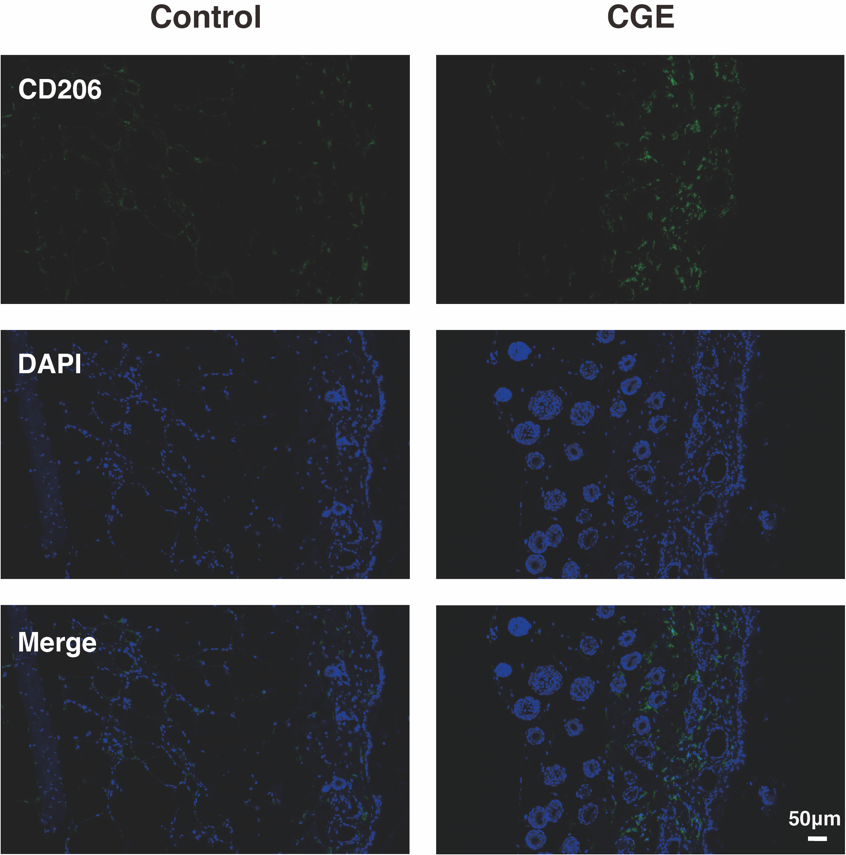
**

**Figure S4.** Immunofluorescence staining of the phenotype of macrophages.

*CGE calvatia gigantea* extract, *CD206* cluster of differentiation 206, *DAPI* 4',6-diamidino-2-phenylindol
